# Antimicrobial and Physicochemical Properties of Hemicellulose-Based Films Incorporating Carvacrol

**DOI:** 10.3390/polym17152073

**Published:** 2025-07-29

**Authors:** Syed Ammar Hussain, Brajendra K. Sharma, Phoebe X. Qi, Madhav P. Yadav, Tony Z. Jin

**Affiliations:** Eastern Regional Research Center, Agricultural Research Service, U.S. Department of Agriculture, 600 E, Mermaid Lane, Wyndmoor, PA 19038, USA; syed.a.hussain@usda.gov (S.A.H.); brajendra.sharma@usda.gov (B.K.S.); phoebe.qi@gmail.com (P.X.Q.); madhav.yadav@usda.gov (M.P.Y.)

**Keywords:** antimicrobial packaging, bio-based films, food safety, micro-emulsions, gas and moisture barrier properties, high-pressure homogenization

## Abstract

Antimicrobial food packaging with natural antimicrobials and biodegradable polymers presents an innovative solution to mitigate microbial contamination, prolong freshness, reduce food waste, and alleviate environmental burden. This study developed antimicrobial hemicellulose-based films by incorporating carvacrol (1% and 2%) as a natural antimicrobial agent through micro-emulsification produced by high-pressure homogenization (M-films). For comparison, films with the same formula were constructed using coarse emulsions (C-films) without high-pressure homogenization. These films were investigated for their antimicrobial efficacy, mechanical and barrier properties, and physicochemical attributes to explore their potential as sustainable antimicrobial packaging solutions. The M-films demonstrated superior antimicrobial activity, achieving reductions exceeding 4 Log CFU/mL against *Listeria monocytogenes*, *Escherichia coli*, and *Salmonella enterica*, compared to the C-films. High-pressure homogenization significantly reduced the emulsion’s particle size, from 11.59 to 2.55 μm, and considerably enhanced the M-film’s uniformity, hydrophobicity, and structural quality. Most importantly, the M-films exhibited lower oxygen transmission (35.14 cc/m^2^/day) and water vapor transmission rates (52.12 g/m^2^/day) than the C-films at 45.1 and 65.5 cc/m^2^/day, respectively, indicating superior protection against gas and moisture diffusion. Markedly improved mechanical properties, including foldability, toughness, and bubble-free surfaces, were also observed, making the M-films suitable for practical applications. This study highlights the potential of high-pressure homogenization as a method for enhancing the functional properties of hemicellulose-based films (i.e., M-films). The fabricated films offer a viable alternative to conventional plastic packaging, paving the way for safer and greener solutions tailored to modern industry needs.

## 1. Introduction

Foodborne diseases and the issue of food waste remain pressing concerns worldwide, affecting public health, threatening food security, and impacting environmental sustainability. According to the CDC, approximately 9.4 million cases of foodborne illness occur each year in the U.S., with frequent offenders including *E. coli* O157:H7, *Salmonella* species, and *Listeria monocytogenes* [1,2]. These pathogens compromise food safety, resulting in substantial economic burdens, estimated at $77.7 billion annually in the U.S. alone [3]. Additionally, food spoilage contributes to 30–40% of global food waste, translating to approximately 1.3 billion tons annually, and generates a carbon footprint of 4.4 gigatons of CO_2_ emissions, making it the third-largest emitter globally [4,5]. These alarming figures underscore the need for innovative packaging solutions to mitigate microbial contamination, extend shelf life, and reduce food waste.

Conventional packaging materials, primarily synthetic plastics, have been indispensable in preserving food quality and safety. However, their resistance to degradation contributes considerably to ecological pollution, necessitating the transition to sustainable alternatives [6,7]. In this context, natural biopolymers such as hemicellulose have garnered attention as eco-friendly substitutes. Hemicellulose, derived from renewable plant biomass, offers biodegradability, renewability, and excellent film-forming capabilities, making it a promising material for food packaging. Despite its advantages, limitations in mechanical strength, barrier properties, and antimicrobial activity restrict its widespread application [8,9,10,11,12,13].

Researchers have explored the incorporation of natural antimicrobial agents into food packaging materials to address these challenges [14,15,16]. Carvacrol, a phenolic compound derived from oregano (*Origanum vulgare*) and thyme (*Thymus vulgaris*) essential oils, is recognized for its potent antimicrobial activity against a broad spectrum of foodborne pathogens and spoilage microorganisms [17,18,19,20], designated as GRAS and authorized for inclusion in food products [21,22,23,24]. Carvacrol has been shown to enhance food safety and extend shelf life when incorporated into biopolymer composite films [18,19,25,26]. However, the effectiveness of carvacrol largely depends on its dispersion within the polymer matrix. Traditional mixing methods often result in uneven distribution, which compromises their effectiveness. Alternatively, micro-emulsification and nano-emulsification techniques, which utilize high-pressure homogenization, have shown promise in uniformly dispersing hydrophobic compounds, such as carvacrol, thereby enhancing film functionality when used as a natural antimicrobial agent [27,28,29].

Recent breakthroughs in biodegradable food packaging highlight the potential of combining hemicellulose with other bio-based materials to optimize functional attributes. For instance, combining hemicellulose with cellulose derivatives has improved mechanical strength and barrier properties [30,31]. Concurrently, films and coatings derived from bio-fiber gum combined with whey protein isolate conjugates demonstrate enhanced physicochemical and antimicrobial properties [32,33]. These efforts align with the principles of intelligent and active packaging systems, designed to extend shelf life and ensure food safety through continuous release mechanisms [34,35].

In our latest studies [30,31], we successfully fabricated hemicellulose-based films that exhibit outstanding mechanical properties, enhanced oxygen barrier performance, robust thermal stability, and favorable physicochemical properties. However, the application of the developed films in antimicrobial packaging has not yet been exploited. Therefore, this study aimed to advance these hemicellulose-based sustainable packaging films to the next level by integrating carvacrol, an essential oil with demonstrated antimicrobial activities. A high-pressure homogenization process was applied to create micro-emulsions before constructing films. The effects of carvacrol concentrations and emulsion process on antimicrobial activities, physicochemical, mechanical properties, and gas permeability of films were investigated.

## 2. Materials and Methods

### 2.1. Materials

Methylcellulose (MC), glycerol (G), and carvacrol (95%, Car) purity were purchased from Millipore Sigma (St. Louis, MO, USA). Pea protein isolate (PPI, unflavored), composed of 69.6% protein, 6.5% fat, and less than 4.3% carbohydrate, was sourced from Swanson^®^, Middletown, PA, USA. High-methoxyl pectin (HMP; Vis-Z Pectin), containing 79.5 ± 2.8% galacturonic acid (GA), 90.8 ± 0.8% degree of methyl esterification (DE), and 26.0 ± 2.4% total sugar (TS), was generously donated by CP Kelco (San Diego, CA, USA). Its composition was analyzed using colorimetric and HPLC methods [30]. Hemicellulose B (HB) was extracted from corn bran in our laboratory following the procedure detailed in our previous publications [30,31,36]. Deionized distilled (dd) water was prepared using a Milli-Q Advantage A10 ultrapure water purification system (EMD Millipore Corporation, Billerica, MA, USA). All chemicals utilized in the study were reagent-grade.

### 2.2. Development of Hemicellulose B (HB)- and Methylcellulose-Based Solutions and Films Incorporating Carvacrol

The composite films were formulated following our initial trials, as described in our recent publications [30,31]. The formulations containing hemicellulose B (HB), methylcellulose (MC), high-methoxyl pectin (HMP), glycerol (G), and pea protein isolate (PPI) were selected (see Table 1) and prepared separately by dispersing the required quantities of each ingredient dissolved in deionized (DI) water at 20 °C. Carvacrol was added to the solutions at the final stage of preparation. Carvacrol was added to the solutions at the final stage of preparation using a volume-to-volume (*v/v*) ratio. This approach follows our previous studies and other references [25,32], as carvacrol essential oil is a volatile liquid. Using a volume basis ensures more accurate dosing and reproducibility. A representative formulation (HB/MC+Car-1%\M) contained 12.0 g of total solids per 100 g of solution, corresponding to a solid content of 12% *w*/*w*. The remaining 88 g was deionized water, and carvacrol was added at a concentration of 1% *v*/*v* (1 mL per 100 mL). The 1% and 2% *v*/*v* concentrations of carvacrol were selected based on preliminary trials and literature reports [25,32], indicating antimicrobial efficacy without compromising film integrity. Higher concentrations (>2%) resulted in phase separation and reduced film homogeneity. The optimum carvacrol ratio is currently under investigation, although initial findings suggest that 2% *v*/*v* provides an optimal balance between antimicrobial activity and mechanical stability.

The film-forming solutions were then formulated according to the ratios and mixing mode in Table 1. The solutions were stirred and degassed using a specialized mixer machine, FlackTek (medium lineup) (FlackTek Manufacturing, Landrum, SC, USA) (ranging from 1500 to 2000 rpm), resulting in coarse emulsions. The coarse emulsions were subjected to high-pressure homogenization (HPH) to create micro-emulsions using an EmulsiFlex-B3 high-pressure homogenizer (Avestin Inc., Ottawa, ON, Canada) at a pressure of 138 MPa (20,000 psi) for two cycles [37].

Following preparation, 30 g of each coarse and micro-emulsion sample were transferred into 100 mm Teflon Petri dishes (Welch Fluorocarbon, Inc., Dover, NH, USA) and subjected to drying in an environmental chamber (Model 7900-33, Caron Scientific, Marietta, OH, USA) set at 20 °C and 50% relative humidity. This process continued for roughly one week or until the resulting film masses stabilized between 1.01 and 1.11 g. After drying, the films were gently removed and placed in Ziploc bags, then stored in desiccators to preserve their properties prior to further analysis [30,31]. Films prepared using coarse emulsions are referred to as the C-film, whereas those produced using micro-emulsions are designated as the M-films.

### 2.3. Particle Size Measurement

The particle size (μm) distribution of the film-casting solutions, as volume (D4, 3), and surface area mean (D3, 2) values, was measured using a Laser Scattering Particle Size Analyzer (Partica LA-950 V2, Horiba Instruments, Inc., Irvine, CA, USA). The solution was diluted with deionized water to achieve proper dispersion before adding it to the analyzer chamber. The volume mean size (μm) (D4, 3) was used for final analysis. Measurements were conducted three times to ensure accuracy, and the average particle size values were recorded [15,37,38].

### 2.4. Film Characterization

#### 2.4.1. Physical Attributes

To facilitate the selection of optimal film-casting formulations, a scoring system was implemented to evaluate the key physical characteristics of the dry films, including peelability, foldability, transparency, and the presence of air bubbles, aiding in the selection of the optimal film-casting composition [30,31]. Peelability was evaluated by observing the ease with which films detached from Petri dishes: those that fractured or failed to detach were rated −2, while those that released smoothly were scored 2. Foldability was determined by gently bending the films at a bilateral angle; films exhibiting major cracks received a −2, whereas those remaining intact were assigned a score of 2. Transparency was evaluated by placing the films adjacent to a placard; films with ambiguous visibility of the sign were given a score of −2, and films showing complete transparency were rated 2. Lastly, the presence of air bubbles was inspected visually, with films exhibiting numerous bubbles assigned a score of −2 and bubble-free films receiving a score of 2. The maximum cumulative score across all evaluated attributes was 8.

#### 2.4.2. Colorimetry Analysis

A digital colorimeter (PCE-CSM 1, PCE Americas Inc., Jupiter, FL, USA) was used to measure the Hunter LAB color parameters of the films: L* (lightness), a* (red-green), and b* (yellow-blue). Each film was placed over a standard white background with reference values of L = 94.52, a = 0.45, and b = 0.04. Measurements were recorded at three randomly selected points per sample. The resulting values were employed to calculate the whiteness index (WI), yellowness index (YI), and total color difference (TCD), as described previously [39].

#### 2.4.3. Film Thickness and Moisture Measurement

Film thickness was measured using a 0–1”/0–25 mm Xtra-Value II Electronic Micrometer (Fowler High Precision, Canton, MA, USA), with three replicate measurements taken per sample to determine average thickness values. Moisture content was assessed using a Torbal ATS 133 Moisture Analyzer (Scientific Industries, Inc., Bohemia, NY, USA) at 120 °C. Initial and final weights of the films were recorded, and the moisture content (%) was calculated using the instrument’s integrated analytical software, Torbal Dryer Manager software V 1.0.

#### 2.4.4. Contact Angle Analysis

Wettability was evaluated by measuring water contact angles using deionized (dd) water as the testing medium. A pocket goniometer (Model PG3, v.3.4; United Testing Systems Inc., New Castle, DE, USA) [30,31], was employed for this analysis. A micropipette was used to dispense 2 μL of water onto the film surface, and the droplet behavior was recorded for 60 s using an integrated video system. Contact angles were determined via the instrument’s software. Measurements were conducted in triplicate at different positions on each sample, and the mean values were reported.

#### 2.4.5. Scanning Electron Microscopy (SEM) Imaging

Film sample images were captured using an FEI Quanta 200F Scanning Electron Microscope (FEI Corporation, Hillsboro, OR, USA), operated in high-vacuum mode with an accelerating voltage of 10 kV. Fractured samples were mounted onto SEM holders using double-sided, electrically conductive carbon-adhesive tape to prevent electrostatic interference during surface morphological analysis [40].

### 2.5. Mechanical Properties

Mechanical testing of the film samples was conducted using a TA-XT plus Texture Analyzer (Stable Micro Systems, Surrey, UK) by the ASTM D882 standard [41] for evaluating tensile properties of film samples. Film specimens measuring 20 mm × 40 mm were prepared and stored in a desiccator containing saturated potassium chloride (KCl) at 22 °C and 50% RH for 48 h prior to testing. Each film strip was initially set at a length of 21 mm and stretched at a constant rate of 2 mm/min. Stress–strain data were recorded using the Exponent software (version 2.64) [30,31].

### 2.6. Permeability Properties

#### 2.6.1. Oxygen Transmission Rate (OTR)

Oxygen transmission rates (OTRs) were determined using an OX-TRAN 1/50 system (MOCON, Minneapolis, MN, USA), following the guidelines outlined in ASTM D3985 [42], as described in prior studies [30,31]. To confine the testing area, an aluminum foil mask with a 5 cm^2^ aperture was utilized. During measurement, nitrogen was supplied to one side of the film while the opposite side was exposed to pure oxygen at 23 °C and 0% relative humidity (RH).

#### 2.6.2. Water Vapor Transmission Rate (WVTR)

Water vapor transmission rates (WVTRs) were determined using the PERMATRAN-W model (MOCON, Minneapolis, MN, USA) in accordance with ASTM E96/E96M standards [43]. Measurements were performed at 23 °C and 50% relative humidity (RH), with results reported in grams per square meter per day (g/m^2^/day) [30,31].

### 2.7. Antimicrobial Properties

#### 2.7.1. Inoculum Preparation

The bacterial strains *Escherichia coli* O157:H7 (ATCC strain 20R2R), *Listeria monocytogenes* (strain 19111), and *Salmonella enterica* (strain 53647, serotype Typhimurium) were obtained from the USDA Eastern Regional Research Center’s culture repository in Wyndmoor, Pennsylvania. Each strain was individually cultured in sterile 50 mL conical tubes containing 30 mL of Tryptic Soy Broth (BBL/Difco, Sparks, MD, USA). The cultures were incubated at 37 °C for 18 h, ensuring optimal growth conditions for subsequent experiments [32,33].

#### 2.7.2. Antimicrobial Properties

The antimicrobial activity of the films was assessed using three methods: agar plate tests with direct contact, agar plate tests with headspace release, and liquid media assays. These procedures were adapted from established protocols [32,33].

For the direct contact assay, film samples with a total surface area of approximately 9 cm^2^ were placed in direct contact with agar plates that had been previously inoculated with bacterial cultures. In the headspace release assay, intact film specimens were positioned on the inner surface of the Petri dish lids, ensuring they avoided direct contact with the agar. All plates were incubated at 25 °C for 24–48 h, after which zones of inhibition around the film samples were measured to assess antimicrobial activity.

In liquid medium tests, four film pieces (total surface area ~9 cm^2^) were immersed in glass tubes containing 9 mL of sterile 0.1% peptone water, which was inoculated with 1 mL of an overnight bacterial culture at an initial population of 10^7^–10^8^ CFU/mL. Tubes were incubated at 25 °C with agitation at 100 rpm. At designated intervals, 1 mL of the solution was sampled and serially diluted with 0.1% peptone water. Then, 100 μL of the appropriate dilutions were plated onto selective agar media. The agar types included Tryptic Soy Agar (TSA) for *E. coli*, PALCAM for *Listeria*, and Xylose Lysine Tergitol 4 Agar (XLT4) for *Salmonella*. Plates were incubated at 37 °C for 24–48 h, depending on the bacterial strain. Control samples consisted of peptone water solutions without films, ensuring reliable comparisons.

### 2.8. Statistical Analysis

All experiments were performed in triplicate, and data were reported as mean ± standard deviation. Statistical comparisons were conducted using one-way ANOVA followed by Tukey’s post hoc test for multiple comparisons, utilizing GraphPad Prism version 7.0 (GraphPad Software, Boston, MA, USA).

## 3. Results and Discussion

In this study, ten hemicellulose-based film formulations were initially prepared using combinations of hemicellulose B (HB), methylcellulose (MC), high-methoxyl pectin (HMP), glycerol (G), pea protein isolate (PPI), and carvacrol. Based on an initial evaluation of their physical attributes, the five most promising films (detailed in Section 2.2 and Table 1) were selected and subjected to thorough investigations.

### 3.1. Particle Size Analysis of the Film-Casting Solutions

**Figure 1:**
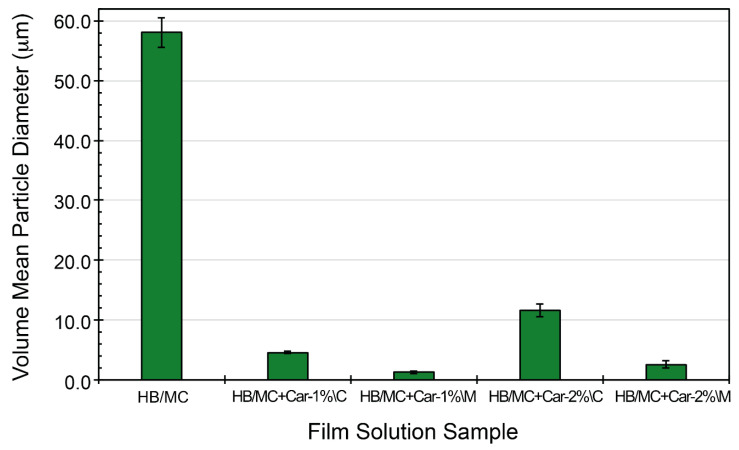
Volume mean particle diameter (μm) of the film-making solution samples studied in this work.

The particle size distribution in the emulsified solutions (used to prepare films) was analyzed to assess the dispersion of carvacrol in the HB/MC system (see Figure 1). The control emulsion (HB/MC), prepared without carvacrol, exhibited the largest average particle size of 58.1 μm. When carvacrol was introduced into the HB/MC system through the coarse emulsification method, the particle sizes were notably reduced, with HB/MC+Car-1%\C and HB/MC+Car-2%\C showing an average particle of 4.56 and 11.59 μm, respectively. The micro-emulsified solutions prepared using high-pressure homogenization demonstrated significantly smaller average particle sizes of 1.23 μm for HB/MC+Car-1%\M and 2.55 μm for HB/MC+Car-2%\M. These findings indicated that the high-pressure homogenization method efficiently reduced the particle size while ensuring uniform dispersion of carvacrol within the HB/MC emulsion system.

These assessments closely aligned with previous studies and demonstrated the reinforcing role of high-pressure homogenization in improving the stability of hydrophobic compounds, such as carvacrol within biopolymer matrices. It has been demonstrated [25,32,37,38] that micro-emulsification enhances the uniformity and stability of active compounds, resulting in films with improved functional properties. The reduced particle size in the M-film casting solutions contributed to an expanded surface area, increased hydrophobicity, and improved uniformity, making them suitable for applications in food packaging where consistent barrier properties are crucial.

### 3.2. Film Characterization

#### 3.2.1. Physical Attributes

**Figure 2:**
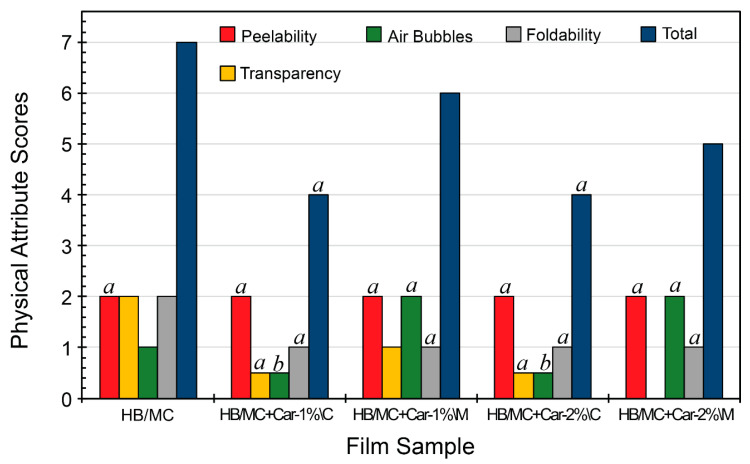
Physical attribute scores of the HB/MC-based film samples. Data sharing the same letter are not statistically significantly different (*p*  >  0.05). Data with no letters are significantly different statistically from other points within the same group. Abbreviations: Hemicellulose B (HB), methylcellulose (MC), carvacrol (Car), coarse emulsion (C), micro-emulsion (M).

Building on previously developed films (HB/MC/HMP/PPI/G) [30,31], this work introduced carvacrol at predefined concentrations, incorporating it using either mixing (coarse emulsions) or high-pressure homogenization (micro-emulsions) (Section 2.2 and Table 1). The physical attributes of the films were assessed using a scoring system that evaluated peelability, foldability, transparency, and air bubble presence to provide a semi-quantitative description of the film quality. Figure 2 illustrates the physical properties, while Figure S1 depicts the films’ visual appearance and transparency.

All tested films exhibited excellent peelability, scoring a maximum of 2, as they detached smoothly from Petri dishes without cracking. Foldability scores varied, with the control film (HB/MC) achieving the highest flexibility (score of 2). In contrast, all C-films and M-films displayed a slight decline in flexibility, likely due to changes in the film matrix resulting from the incorporation of carvacrol.

Transparency was highest in the control film (HB/MC: score of 2) and slightly lower for the C-films, which earned a score of 0.5. In contrast, the M-film (HB/MC+Car-1%\M) scored 1, whereas HB/MC+Car-2%\M scored zero due to microstructural modifications introduced during emulsification, which significantly reduced transparency. Air bubble formation was a key differentiator between methods. The control film displayed a minor presence of air bubbles, while the C-films exhibited considerable presence of air bubbles, resulting in scores of 0.5. In contrast, the M-films were bubble-free and earned the highest score of 2, indicating improved uniformity by the micro-emulsification process.

Overall, the total scores highlighted the differences among films. The control film and the M-films achieved a higher total score than the C-films, demonstrating superior physical attributes. Conversely, the C-films scored 4 due to the presence of air bubbles and reduced foldability (Figure 2). The findings suggest that emulsification enhances the uniformity and quality of hemicellulose-based films, albeit at the expense of a marginal compromise in transparency. By improving foldability and achieving bubble-free films, carvacrol-incorporated M-films exhibit properties that make them highly suitable for practical packaging applications (Figure 1 and Figure 2).

#### 3.2.2. Film Color

The color properties of all hemicellulose-based films were evaluated using digital colorimetry to assess their lightness (L), redness-greenness (a), and yellowness-blueness (b*). Table 2 presents the color values of various hemicellulose-based film samples, including the whiteness index (WI), yellowness index (YI), and total color difference (TCD). The control film demonstrated moderate lightness (L = 85.92), with a slightly red-green and yellow hue (a = 2.10, b = 14.86).

Incorporating carvacrol altered the film’s color characteristics. The C-films exhibited reduced lightness and increased yellowness compared to the control. For example, HB/MC+Car-1%\C showed L = 84.72 and b = 16.24, while HB/MC+Car-2%\C demonstrated even lower lightness (L = 83.98) and higher yellowness (b = 17.14). On the other hand, the M-films exhibited comparable lightness and higher yellowness. Specifically, HB/MC+Car-1%\M retained L = 85.76 and b = 15.84, while HB/MC+Car-2%\M exhibited L = 85.60 and b = 16.28.

The variations in total color difference (TCD) values further highlighted the distinction between the methods. The M-films consistently showed lower TCD values than the C-films, reflecting fewer color alterations. For example, the TCD for HB/MC+Car-1%\M was 9.24, significantly lower than the 15.72 observed for HB/MC+Car-1%\C, indicating reduced color alteration due to the uniform dispersion of carvacrol through emulsification.

The observed differences in color properties were attributed to the method of carvacrol incorporation and its impact on the uniformity of the polymer matrix. The C-films prepared from the coarse emulsion demonstrated greater color changes, likely due to the uneven distribution of carvacrol, which creates regions of optical heterogeneity. Consistent with Nunes et al. (2023), including additives led to noticeable visual alterations, especially in less homogenized systems [39].

On the other hand, the M-films showed enhanced color alteration, retaining higher lightness and lower TCD values. This reduction suggests that emulsification ensured a more uniform dispersion of carvacrol, minimizing visual discrepancies. Krümmel et al. (2024) observed comparable effects in cassava starch films containing carvacrol nano-capsules, where uniform dispersion via emulsification significantly reduced color differences and improved visual consistency [25].

These findings are particularly relevant for food packaging applications where the visual appeal of films is essential for consumer acceptance. Transparent or semi-transparent films benefit from maintaining consistent lightness and minimal color changes. Moreover, the enhanced compatibility between carvacrol and hemicellulose achieved through emulsification improved both esthetic properties and functional attributes, as demonstrated by the improved antimicrobial and barrier performance observed in other parts of the current study.

The M-films exhibited lower transparency levels relative to both the control and the C-film counterparts, which aligned well with the slight alterations in optical properties observed through colorimetric analysis. However, the high-pressure homogenization enhanced structural uniformity, as evidenced by the absence of air bubbles, which correlates with higher color alterations. These findings highlight that while micro-emulsification may slightly compromise transparency, it proves advantageous in achieving consistent visual and structural quality, making the M-films well suited for practical applications that require esthetic reliability and functional applicability.

#### 3.2.3. Film Thickness and Moisture Content

The thickness of hemicellulose-based films is a critical parameter that influences their mechanical strength and structural integrity [44,45]. Table 2 presents the thickness measurements (μm) of the HB/MC-based films. The data demonstrated slight variations across formulations due to the incorporation of carvacrol and processing techniques. The control film exhibited a thickness of 212 μm, while C-films (HB/MC+Car-1%\Cand HB/MC+Car-2%\C) showed increased thicknesses of 214 and 217 μm, respectively. Similarly, the M-films (HB/MC+Car-1%\M and HB/MC+Car-2%\M) displayed slightly greater thickness of 216 and 219 μm, respectively. However, these differences were not statistically significant. These increases were attributable to the structural impact of carvacrol addition and the role of high-pressure homogenization in enhancing matrix dispersion, thereby influencing the final film composition.

**Figure 3:**
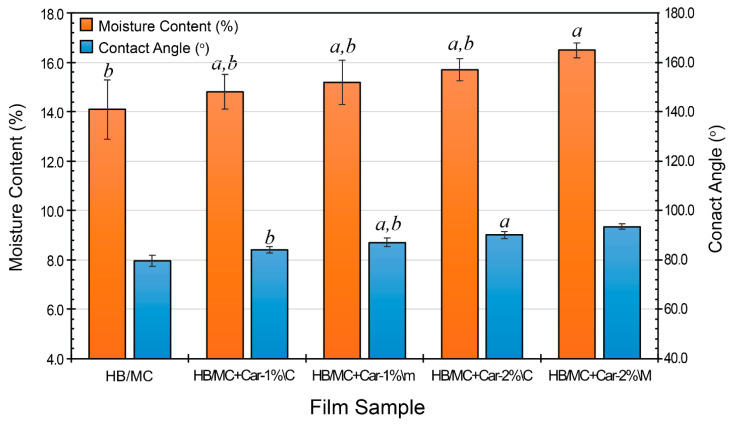
Moisture content (%) and contact angle (^o^) of the HB/MC-based film samples. Data are mean  ±  standard deviation (*n*  =  3). Data sharing the same letter are not statistically significantly different (*p  >*  0.05). Data with no letters are statistically significantly different from other points within the same group.

Moisture content is a major factor in film stability and barrier efficiency. Figure 3 shows a similar trend to that of film thickness (Table 2), with incremental increases corresponding to the carvacrol concentration. The control film had a moisture content of 14.1%, whereas films containing carvacrol displayed higher values at 14.8 and 15.7% for the C-films (HB/MC+Car-1%\C and HB/MC+Car-2%\C), and 15.2 and 16.5% for the M-films (HB/MC+Car-1%\M and HB/MC+Car-2%\M). These increases were statistically significant compared to the control, indicating possible hydrophilic interactions between carvacrol molecules and the film matrix during preparation.

These observations correlated carvacrol incorporation methods with the film thickness and moisture content. Films prepared through the micro-emulsification method demonstrated slightly better uniformity and moisture retention than those obtained from the coarse emulsion method, aligning with previous studies that have shown micro-emulsification to be an effective technique for integrating bioactive additives into polymer matrices [46,47]. These successful attempts demonstrated that fine-tuning the film composition and production method improved barrier functionality, thereby reinforcing its effectiveness in maintaining product integrity and prolonging shelf life.

#### 3.2.4. Contact Angle Analysis

The surface wettability of hemicellulose-based films was evaluated through contact angle measurements, as shown in Figure 3, to determine their interaction with aqueous media. The control film exhibited a contact angle of 79.6°, indicating moderate hydrophobicity. The control film had a contact angle of 79.6°, which indicated moderate hydrophobicity. The inclusion of carvacrol considerably improved the films’ hydrophobicity. This rise may have occurred because carvacrol, a hydrophobic compound, migrated to the film’s surface during drying, resulting in a more water-repellent outer layer. C-films containing 1% and 2% carvacrol (HB/MC+Car-1%\C and HB/MC+Car-2%\C) showed contact angles of 84.1° and 90.1°, respectively, while the M-films (HB/MC+Car-1%\M and HB/MC+Car-2%\M) demonstrated even higher hydrophobicity, with contact angles of 87.1° and 93.4°. These statistically significant differences supported that increasing carvacrol concentration and micro-emulsification enhanced the film’s hydrophobicity. The superior hydrophobic performance of the M-films underscores the effectiveness of this technique in uniformly dispersing carvacrol within the polymer matrix.

These results agreed with previous studies indicating that incorporating hydrophobic agents enhanced the water resistance and surface properties of biopolymer films [37,47,48,49]. The increased hydrophobicity of the M-films reduced their wettability, making them particularly suitable for food packaging applications.

#### 3.2.5. Film Morphology and Microstructure

**Figure 4:**
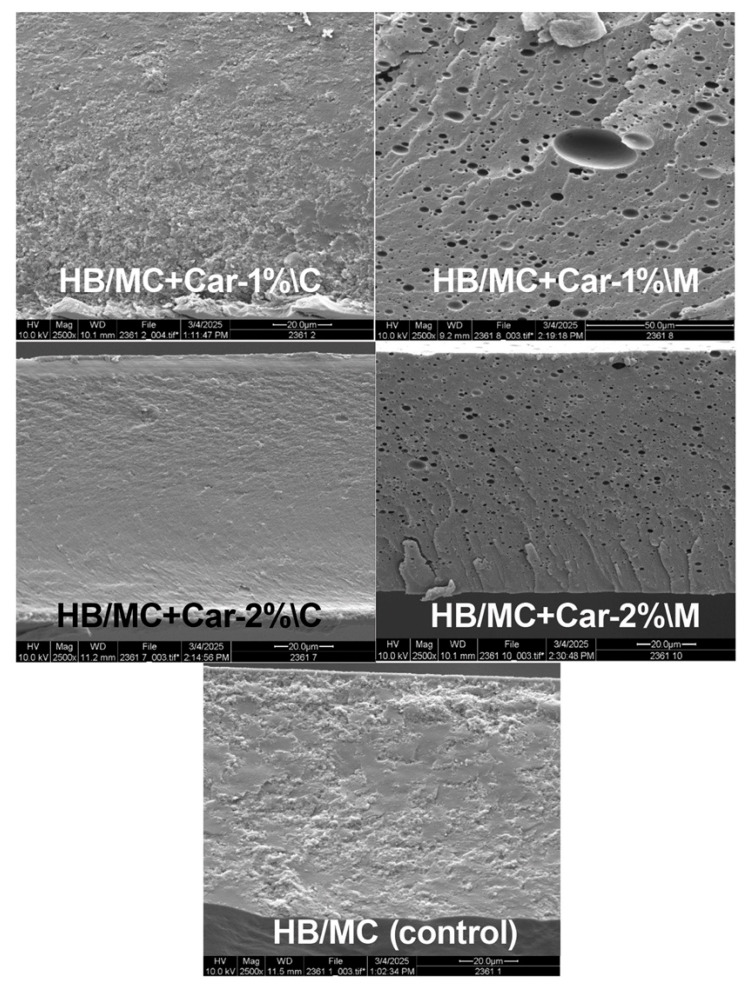
SEM images of the HB/MC films studied in this work.

SEM analysis provided offered high-resolution visualization of the microstructure of hemicellulose-based films. Figure 4 highlights the microscale features and their implications for film functionality. The SEM images of the control film showed a relatively smooth surface, indicating a uniform distribution of polymers. However, slight aggregation and discontinuities were observed, which may be due to the absence of carvacrol and the less cohesive network formation. These structural characteristics were associated with moderate tensile strength and elongation values measured in mechanical properties. The control film exhibited limited compatibility between hemicellulose and other components, resulting in decreased structural integrity and barrier performance (as discussed in Section 3.4 below).

In contrast, the M-films demonstrated a remarkably smooth and homogeneous microstructure. The SEM images revealed a compact film surface with uniformly dispersed carvacrol particles (small dark holes) throughout the hemicellulose matrix. This uniform distribution can be attributed to the effectiveness of the emulsification process, which promoted stronger intermolecular interactions and enhanced the structural cohesiveness of the film. The smoother topography was directly correlated with improved mechanical properties, such as greater elongation and toughness (Section 3.3). Additionally, this uniformity contributed to enhanced barrier properties, as reflected by the reduced OTR and WVTR values (Section 3.4). The finer morphological structure of the M-films suggests improved compatibility between hemicellulose and carvacrol, facilitated by the emulsification technique. This synergistic effect reinforced the polymer matrix, minimizing pathways for moisture penetration and gas diffusion. These observations were consistent with previous studies [50,51,52], which reported that well-dispersed bioactive compounds significantly enhanced the structural integrity and functional attributes of biopolymer films. Furthermore, the compact and cohesive structure of the M-films supports their role as effective oxygen and moisture barriers, making them suitable for food packaging applications requiring high durability and protection against environmental factors.

### 3.3. Mechanical Properties

**Figure 5:**
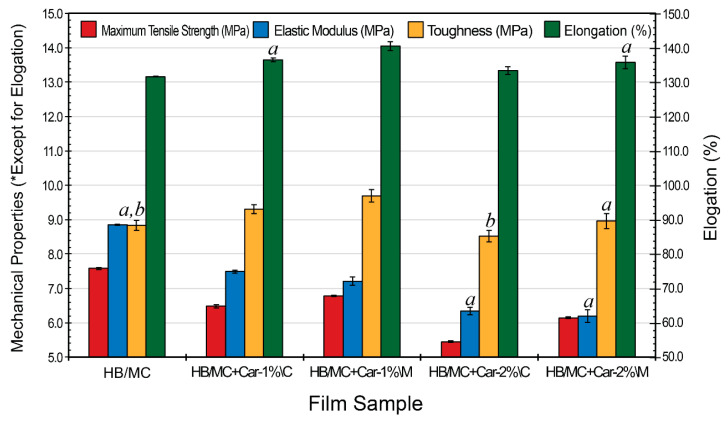
Mechanical properties for the HB/MC-based film samples. Data are mean  ±  standard deviation (*n*  =  3). Data sharing the same letter are not statistically significantly different (*p  >*  0.05). Data with no letters are statistically significantly different from other points within the same group.

Figure 5 presents the mechanical performance metrics of hemicellulose-based films, encompassing maximum tensile strength, elongation at break, elastic modulus, and toughness. The integration of carvacrol led to a slight reduction in tensile strength the peak force the film can resist before rupture, suggesting a minor compromise in structural integrity associated with its incorporation. The control film exhibited the highest tensile stress of 7.58 MPa, while the C-films (HB/MC+Car-1%\C and HB/MC+Car-2%\C) showed lower values of 6.48 and 5.45 MPa, respectively. In comparison, the M-films (HB/MC+Car-1%\M and HB/MC+Car-2%\M) achieved slightly improved tensile stresses of 6.78 and 6.15 MPa, suggesting that micro-emulsification partially mitigated the reduction in strength.

Elongation at break, an indicator of the film’s flexibility, increased with the incorporation of carvacrol. The C-films (HB/MC+Car-1%\C and HB/MC+Car-2%\C) exhibited elongations of 136.5% and 133.4%, respectively, compared to the control film at 131.6%. The M-films exhibited the highest elongation values of 140.5% (1% carvacrol) and 135.7% (2% carvacrol), highlighting the ability of emulsification to enhance flexibility by promoting a more uniform distribution of carvacrol.

The elastic modulus, reflecting the films’ rigidity, declined upon the addition of carvacrol, suggesting a reduction in structural stiffness associated with its incorporation. The control film displayed the highest modulus of 8.85 MPa, while the C-films (HB/MC+Car-1%\C and HB/MC+Car-2%\C) recorded reduced values at 7.49 and 6.35 MPa, respectively. The M-films showed further reductions in modulus at 7.21 and 6.19 MPa, reflecting increased flexibility due to structural alterations induced by emulsification.

Toughness, which reflects the film’s ability to absorb energy before breaking, improved with the incorporation of carvacrol. The C-films (HB/MC+Car-1%\C and HB/MC+Car-2%\C) reached 9.31 and 8.52 MPa, respectively, compared to the control film at 8.83 MPa. The M-films demonstrated the highest toughness at 9.69 and 8.96 MPa, indicating superior energy absorption capabilities associated with the emulsification process.

These findings are consistent with prior research on hemicellulose-based films. Ahmadi et al. (2012), for instance, documented elongation values ranging from 13% in ethylene–vinyl alcohol (EVOH) films to 64% in films derived from sugarcane bagasse [52,53]. In comparison, the HB/MC and HB/CMC formulations developed in this study achieved markedly higher elongation values of 131% and 139%, respectively, demonstrating a substantial improvement in film flexibility. Additionally, the toughness values of 8.83 and 9.69 MPa in this study also exceeded those reported for other biopolymer films [54,55], demonstrating the benefits of incorporating plasticizers and bioactive compounds, such as carvacrol. “The enhanced mechanical performance is likely due to the molecular compatibility between hemicellulose and co-formulated components, facilitated by intermolecular hydrogen bonding that promotes cohesive film structure [56,57]. These non-covalent interactions, promoted by plasticizers, further enhanced these properties, consistent with studies by Vanin et al. [58] and Tabari [59]. In contrast, films lacking additional additives showed only moderate mechanical performance, likely due to poor adhesion among the polymer components [60,61].

In conclusion, the application of micro-emulsification significantly improved the mechanical performance of hemicellulose-based films, underscoring its potential as a valuable technique for optimizing biofilm properties in packaging and related applications. The improved flexibility, toughness, and energy absorption characteristics make these films highly suitable for packaging applications. Future studies should investigate the role of cross-linking (non-covalent) agents and advanced emulsification techniques to further refine mechanical performance.

### 3.4. Permeability Properties

#### 3.4.1. Oxygen Transmission Rate (OTR)

**Figure 6:**
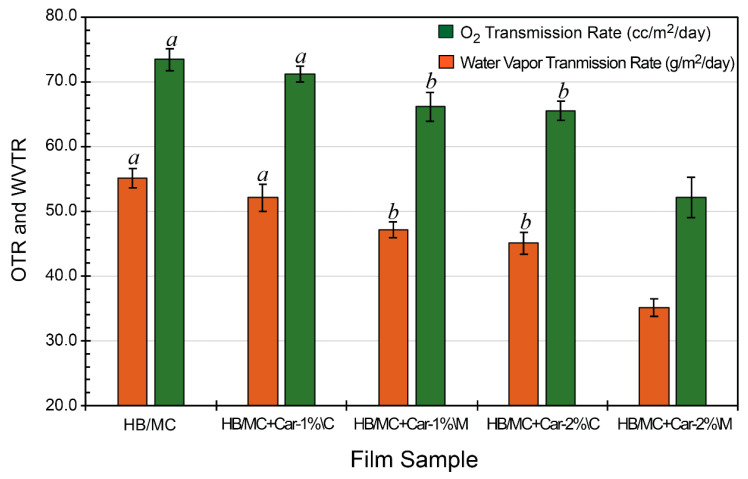
Oxygen (OTR) and water vapor transmission rate (WVTR) of the HB/MC-based film samples (w/wo carvacrol). Data are mean  ±  standard deviation (*n*  =  3). Data sharing the same letter are not statistically significantly different (*p  >*  0.05). Data with no letters are statistically significantly different from other points within the same group.

As depicted in Figure 6, the oxygen transmission rate (OTR) of hemicellulose-based films was measured to evaluate their barrier efficiency against oxygen permeation. The control film exhibited an OTR at 55.1 cc/m^2^/day, indicating moderate oxygen permeability. Incorporating carvacrol into the films significantly reduced OTR, suggesting enhanced oxygen barrier properties. The C-films showed OTR values of 52.1 cc/m^2^/day for HB/MC+Car-1%\C and 45.1 cc/m^2^/day for HB/MC+Car-2%\C. On the other hand, the M-films exhibited superior performance, with OTR values of 47.2 cc/m^2^/day and 35.1 cc/m^2^/day for HB/MC+Car-1%\M and HB/MC+Car-2%\M, respectively. The HB/MC+Car-2%\M film exhibited a remarkably low oxygen transmission rate (OTR), approximately 35 cc/m^2^/day, comparable to biodegradable films such as poly(hydroxybutyrate) and poly(3-hydroxybutyrate-co-3-hydroxy-valeriate), which exhibit OTR values around 23 cc/m^2^/day [54,62]. Additionally, these films offered substantial environmental benefits by repurposing agricultural waste, reducing waste output, increasing resilience, and providing antimicrobial efficacy.

The enhanced oxygen barrier performance observed in the M-films is likely due to the more homogeneous distribution of carvacrol throughout the polymer matrix, which may have contributed to reduced diffusion pathways for oxygen molecules. This uniformity effectively minimized oxygen diffusion pathways, enhancing the films’ barrier properties. The particle size analysis (Section 3.1) further supported this observation, as the M-films were prepared from solutions that achieved significantly smaller and more consistent particle sizes. This uniformity likely improved the films’ structural density, correlating with better oxygen barrier properties.

These findings align with prior research demonstrating that the uniform incorporation of hydrophobic bioactive compounds can effectively reduce oxygen permeability in biopolymer-based films [25,37,38]. The enhanced barrier properties achieved through emulsification underscored its potential for advanced food packaging applications where oxygen-sensitive products require protection [15,63]. By enhancing particle dispersion uniformity and reducing permeability, emulsification proved to be a promising strategy for tailoring the physical and functional characteristics of hemicellulose-based films [18,64].

#### 3.4.2. Water Vapor Transmission Rate (WVTR)

The water vapor transmission rate (WVTR) of hemicellulose-based films was measured to evaluate their effectiveness as a moisture barrier, as shown in Figure 6. The control film exhibited a WVTR of 73.5 g/m^2^/day, indicating moderate resistance to moisture transmission. Incorporating carvacrol significantly decreased WVTR, demonstrating enhanced moisture barrier properties. The C-films, HB/MC+Car-1%\C and HB/MC+Car-2%\C, showed improved WVTR values of 71.2 and 65.5 g/m^2^/day, respectively. The M-films, HB/MC+Car-1%\M and HB/MC+Car-2%\M, with WVTR values of 66.2 and 52.1 g/m^2^/day, significantly surpassed these already-improved barrier parameters.

The enhanced moisture barrier properties of the M-films can be attributed to their more homogeneous structure, as evidenced by the particle size analysis (Section 3.1) and the SEM images (Section 3.2.5). These analyses concurred with prior studies, which have shown that a more uniform dispersion of the hydrophobic bioactive compounds (e.g., carvacrol) can significantly enhance the water vapor barrier properties of biopolymer films [15,18,25,32,33,63,65]. The superior WVTR performance of the M-films highlighted their potential for applications in food packaging, particularly for products requiring controlled moisture barriers to maintain quality and shelf life.

The OTR and WVTR of the hemicellulose-based films shared a similar dependence on particle size and the method used to incorporate hydrophobic agents, such as carvacrol. The M-films exhibited lower OTR and WVTR values than the C-films, possibly due to the smaller and more uniformly dispersed particles produced by the high-pressure, homogenization-induced micro-emulsions. This process ensures that carvacrol is evenly distributed within the hemicellulose matrix, resulting in smaller particles that fill voids more effectively and create a denser, less porous film structure. This denser structure minimizes pathways for the diffusion of oxygen and water vapor. In contrast, the coarse emulsion method often produces larger, unevenly distributed particles, resulting in voids and micro-gaps within the film matrix. These gaps increased oxygen and moisture permeability, leading to higher OTR and WVTR values. The relationship between particle size, uniform dispersion, and barrier properties highlights micro-emulsification as a superior method for enhancing oxygen and moisture resistance in hemicellulose-based films, thereby making them more suitable for packaging applications that demand strong barrier performance.

### 3.5. Antimicrobial Properties

In our preliminary investigations, the antibacterial efficacy of the film samples was evaluated using an in vitro method of agar plates. Figure S2A,B show two methods used in this work: direct contact (film samples placed on the agar surface) and indirect contact (film samples placed on the Petri dish lid). All Petri plates were incubated at 37 °C for 24 to 48 h. After the incubation period, the carvacrol-containing film samples exhibited a distinct zone of inhibition compared to the controls, indicating the antibacterial properties of the film samples (Figure S2).

**Figure 7:**
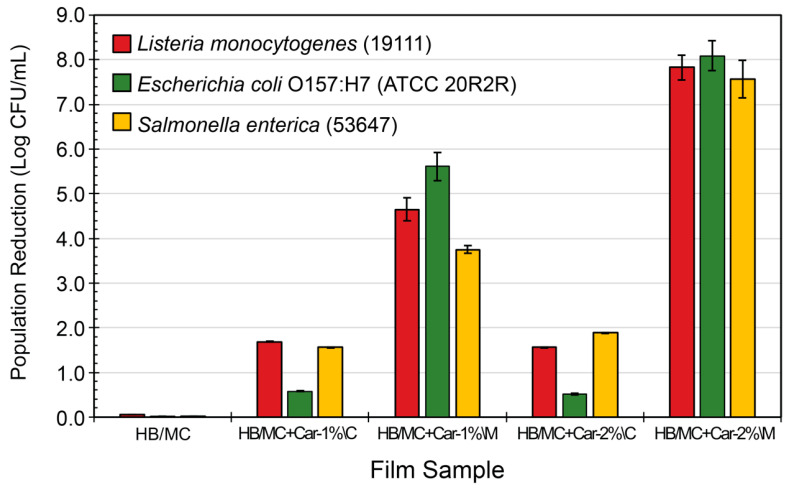
Inhibitory activity of the HB/MC-based film samples against common foodborne pathogens *Listeria monocytogenes* (19111), *Escherichia coli* O157:H7 (ATCC 20R2R), and *Salmonella enterica* (53647). Data are presented as the average of three sets of independent experiments, with each set replicated in duplicate.

For the peptone solution test, the antimicrobial activity of all hemicellulose-based films was also studied. The films were evaluated against three significant foodborne pathogens: *Listeria monocytogenes* (strain 19111), *Escherichia coli* O157:H7 (ATCC strain 20R2R), and *Salmonella enterica* (strain 53647, serotype Typhimurium). Figure 7 shows the reductions in microbial populations (expressed in Log CFU/mL). The control films contained no carvacrol and exhibited minimal antimicrobial activity, achieving reductions of only 0.05, 0.02, and 0.01 Log CFU/mL for *L. monocytogenes*, *E. coli*, and *Salmonella*, respectively.

Incorporating carvacrol significantly enhanced the films’ antimicrobial performance, with the M-films consistently demonstrating higher efficacy than the C-films. For *L. monocytogenes*, the M-films containing 1% carvacrol (HB/MC+Car-1%\M) displayed a reduction of 4.65 Log CFU/mL. In contrast, the C-films achieved a reduction of only 1.68 Log CFU/mL at the same concentration (HB/MC+Car-1%\C). Increasing carvacrol concentration to 2% further improved antimicrobial activity, with the M-films (HB/MC+Car-2%\M) achieving a reduction of 7.83 Log CFU/mL, while the C-films (HB/MC+Car-2%\C) exhibited a reduction of 1.56 Log CFU/mL.

Similar results were obtained for *E. coli*, where the M-films with 1% and 2% carvacrol (HB/MC+Car-1%\M and HB/MC+Car-2%\M) arrived at 5.61 and 8.09 Log CFU/mL reductions, respectively, compared to much lower reductions of 0.58 and 0.51 Log CFU/mL for their C-films counterparts (HB/MC+Car-1%\C and HB/MC+Car-2%\C) (Figure 7). For *Salmonella*, the M-films demonstrated superior antimicrobial performance, reaching reductions of 3.75 and 7.56 Log CFU/mL for 1% and 2% carvacrol concentrations (HB/MC+Car-1%\M and HB/MC+Car-2%\M, respectively). On the other hand, the C-films with the same concentrations exhibited reductions of only 1.56 and 1.88 Log CFU/mL.

The superior antimicrobial activity of the M-films stems from the homogeneous dispersion of carvacrol within the hemicellulose matrix. Micro-emulsification of the film solution facilitates the homogenous incorporation of carvacrol, enhancing its availability for interaction with microbial cells. This mechanism aligns with the findings by Guo et al. (2015), demonstrating that edible films prepared using micro-emulsions exhibit improved antimicrobial properties due to enhanced dispersion of bioactive compounds [37,38]. Jin et al. (2022) [15] also noted that encapsulating essential oils, such as carvacrol, within polymer matrices allows for controlled release, thereby prolonging their antimicrobial activity and ensuring consistent efficacy against microbial populations.

The antimicrobial activity of carvacrol is likely mediated by its ability to disrupt bacterial cell membranes, resulting in increased permeability, leakage of intracellular contents, and ultimately, cell lysis. Carvacrol enters the microbial cell undissociated and ionizes in the cytoplasm’s alkaline environment, releasing hydrogen ions. This process lowers intracellular pH, causing structural damage to proteins, DNA, and extracellular membranes, which disrupts enzymatic functions and ultimately leads to cell death [15,66,67]. Vylkova [63] highlighted similar pH modulation effects in pathogenic fungi, emphasizing the broad antimicrobial potential of carvacrol.

Additionally, the enhanced structural properties of the M-films, demonstrated in SEM micrographs, contribute to their overall performance. The compact and cohesive structure achieved through high-pressure homogenization strengthens the film matrix, thereby reducing oxygen and moisture permeability and indirectly supporting antimicrobial efficacy. Krümmel et al. (2024) [25] observed similar improvements in physicochemical properties when carvacrol nano-capsules were incorporated into cassava starch-based films. Jin et al. [15,32,33] further noted that bioactive agents dispersed uniformly in polysaccharide–whey protein matrices resulted in synergistic improvements in antimicrobial and barrier properties.

These findings are consistent with previous studies [15,25,37,38], where bio-fiber gum (BFG) composite films combined with allyl isothiocyanate (AIT) reduced microbial counts on food surfaces and within in vitro systems by over 4 and 5 Log CFU/mL, respectively. Due to its less pungent odor, the replacement of AIT with carvacrol demonstrated comparable efficacy, validating carvacrol’s suitability for food safety applications.

Overall, the M-films containing carvacrol achieved microbial reductions exceeding 4 Log CFU/mL for *L. monocytogenes* and *E. coli*, meeting industrial benchmarks for antimicrobial packaging materials. The results showcased micro-emulsification as a transformative approach for incorporating bioactive compounds, ensuring uniform dispersion and maximizing antimicrobial potential. Future research should explore additional bioactive agents and optimize micro-emulsification parameters to target a broader spectrum of pathogens and expand scalability for commercial applications.

## 4. Conclusions

This study represented a significant breakthrough in developing hemicellulose-based biodegradable and antimicrobial film formulation and materials. With exceptional antimicrobial efficacy, superior oxygen and water vapor barrier properties, phenomenal structural uniformity, consistent color stability, and remarkable mechanical properties, these films have the potential to significantly advance food packaging technologies. Additionally, the high-pressure-induced micro-emulsification process proved highly effective and advantageous in homogeneously distributing antimicrobial compounds (often hydrophobic) for subsequent film construction. Among the tested films, the M-films with carvacrol concentrations of 1 and 2% demonstrated microbial reductions exceeding 4 Log CFU/mL, oxygen transmission rates of 35.1 cc/m^2^/day, and water vapor transmission rates of 52.1 g/m^2^/day.

Despite these promising results, certain limitations remain. Firstly, the scalability of the micro-emulsification process requires further evaluation to ensure cost-effectiveness and industrial feasibility. Secondly, this study did not fully explore the films’ performance under extreme environmental conditions, such as high humidity or temperature fluctuations. Lastly, and most importantly, antimicrobial testing should be expanded to include a broader spectrum of microorganisms, including fungi, which could further validate the films’ protective potential. Future research should optimize the micro-emulsification process by refining homogenization parameters, such as pressure and number of passages, to improve efficiency and reduce energy consumption. Long-term biodegradability studies are also essential to comprehensively evaluate the environmental impact of these films, ensuring their degradation into harmless byproducts under real-world conditions. Additionally, other natural bioactive compounds should be incorporated into the film matrix to enhance their functional versatility and broaden their applicability across various food categories. This study represents a critical step toward developing high-performance, eco-friendly antimicrobial packaging materials that address key challenges in food safety and environmental sustainability.

## Figures and Tables

**Table 1:** Film samples studied and their compositions (% *w*/*w*, unless otherwise noted).

Film Sample	HB	MC	Pectin (HMP)	Pea Protein Isolate (PPI)	Glycerol (G)	Carvacrol (%, *v*/*v*)	Mode of Mixing
HB/MC (control)	88.4	10	0.1	0.5	1.0	0.0	Coarse emulsification
HB/MC+Car-1%\C	88.4	10	0.1	0.5	1.0	1.0	Coarse emulsification
HB/MC+Car-1%\M	88.4	10	0.1	0.5	1.0	1.0	Micro-emulsification
HB/MC+Car-2%\C	88.4	10	0.1	0.5	1.0	2.0	Coarse emulsification
HB/MC+Car-2%\M	88.4	10	0.1	0.5	1.0	2.0	Micro-emulsification

**Table 2:** Color analysis and thickness measurements of the HB/MC-based film samples studied in this work.

Film Sample	L*	a*	b*	Whiteness Index	Yellowness Index	Total Color Difference (TCD)	Thickness (μm)
HB/MC (control)	85.92 ± 0.18	2.10 ± 0.02	14.86 ± 0.11	80.14 ± 0.89	24.78 ± 0.88	0.00 ± 0.00	212 ± 1.21
HB/MC+Car-1%\C	84.72 ± 0.22	2.34 ± 0.01	16.24 ± 0.13	78.12 ± 0.84	26.43 ± 0.91	15.72 ± 0.41	214 ± 1.34
HB/MC+Car-1%\M	85.76 ± 0.16	2.08 ± 0.02	15.84 ± 0.12	79.52 ± 0.82	25.92 ± 0.87	9.24 ± 0.36	216 ± 1.28
HB/MC+Car-2%\C	83.98 ± 0.25	2.46 ± 0.01	17.14 ± 0.15	77.24 ± 0.87	28.12 ± 0.94	21.30 ± 0.59	217 ± 1.47
HB/MC+Car-2%\M	85.60 ± 0.20	2.12 ± 0.02	16.28 ± 0.14	79.38 ± 0.85	26.14 ± 0.90	12.54 ± 0.48	219 ± 1.39

* Data are presented as mean (*n* = 3) ± standard deviation.

## Data Availability

Data is contained within the article.

## Supplementary Materials

The following supporting information can be downloaded at: https://www.mdpi.com/article/10.3390/polym17152073/s1. Figure S1. Photographs of the HB/MC-based film samples studied in this work. Figure S2. Selected film samples illustrate the two methods used in this work to determine the antimicrobial activities of the HB/MC-based films against common foodborne pathogens. A: direct surface contact; B: film on lid (headspace release).
