# Supplementary material for: Antimicrobial and Physicochemical Properties of Hemicellulose-Based Films Incorporating Carvacrol
Source: Polymers (Basel). 2025 Jul 29;17(15):2073. doi: 10.3390/polym17152073 (PMC12349191; doi:10.3390/polym17152073)
Supplement: Supplementary file 1 [file polymers-17-02073-s001.zip › polymers-3760771-supplementary.pdf]

Supplementary Material

Antimicrobial and Physicochemical Properties of Hemicellulose-Based Films Incorporating Carvacrol

Syed Ammar Hussain, Brajendra K. Sharma, Phoebe X. Qi, Madhav P. Yadav, Tony Z. Jin*

Eastern Regional Research Center, Agricultural Research Service, U. S. Department of Agriculture
600 E. Mermaid Lane, Wyndmoor, PA 19038, USA

*Corresponding author:
Dr. Tony Z. Jin
TEL: 215-836-6904
FAX: 215-233-6406
E-mail: tony.jin@usda.gov

Mention of trade names or commercial products in this publication is solely for the purpose of providing specific information and does not imply recommendation or endorsement by the U.S. Department of Agriculture (USDA). The USDA is an equal opportunity provider and employer.

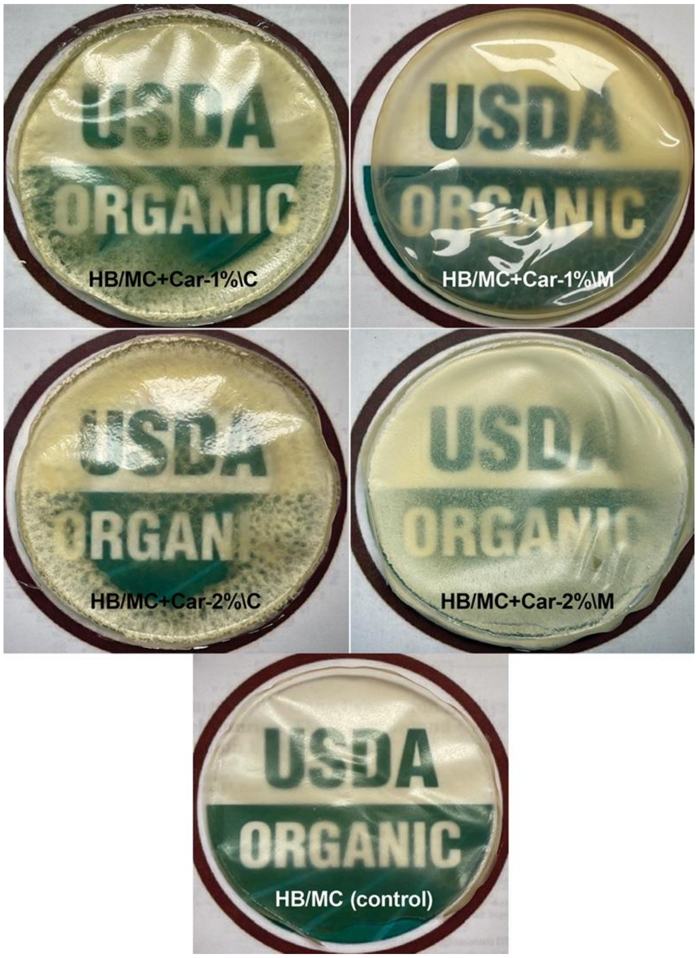

Figure S1. Photographs of the HB/MC-based film samples studied in this work.

A

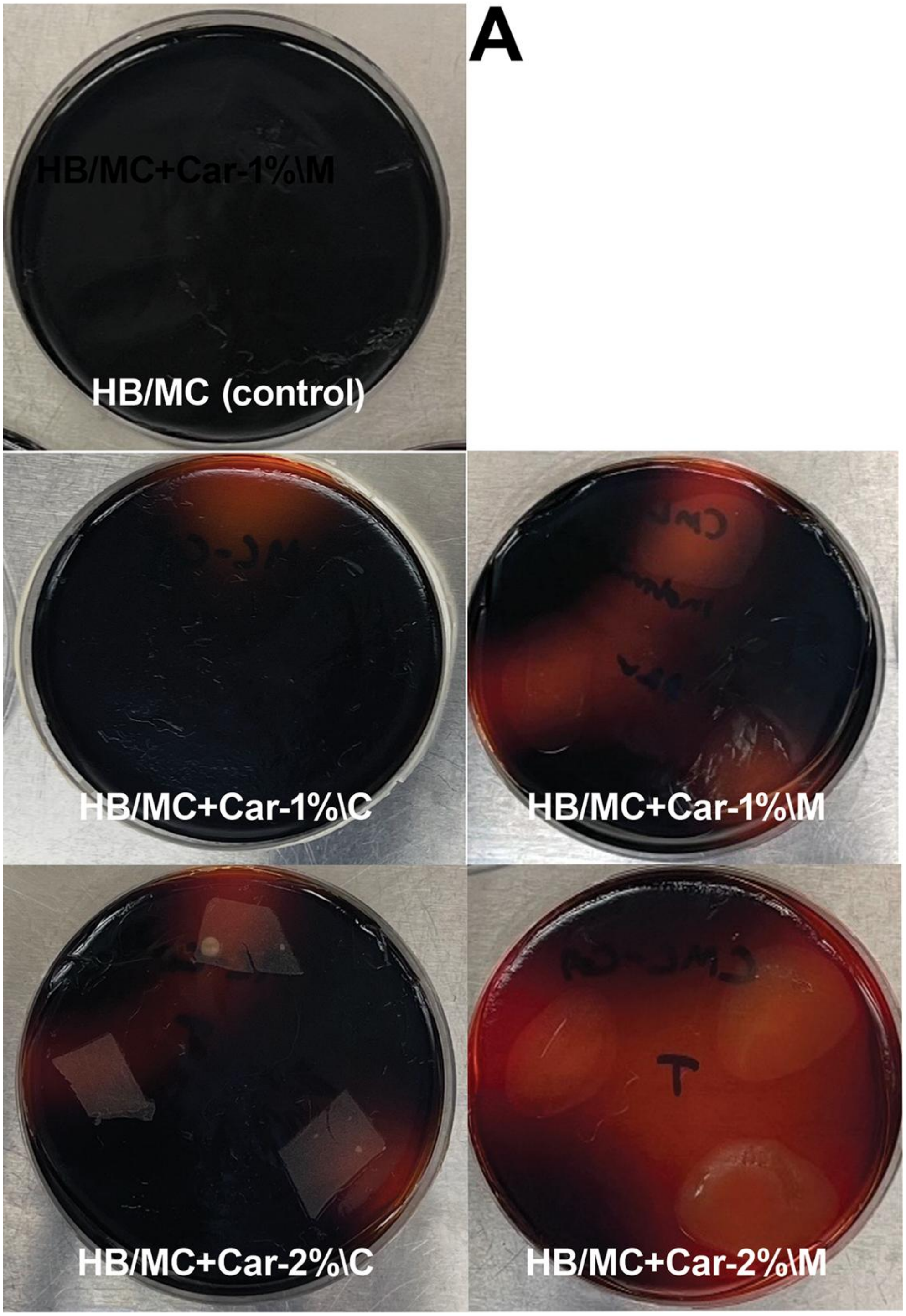

HB/MC+Car-1%\M
HB/MC (control)
HB/MC+Car-1%\C
HB/MC+Car-1%\M
HB/MC+Car-2%\C
HB/MC+Car-2%\M

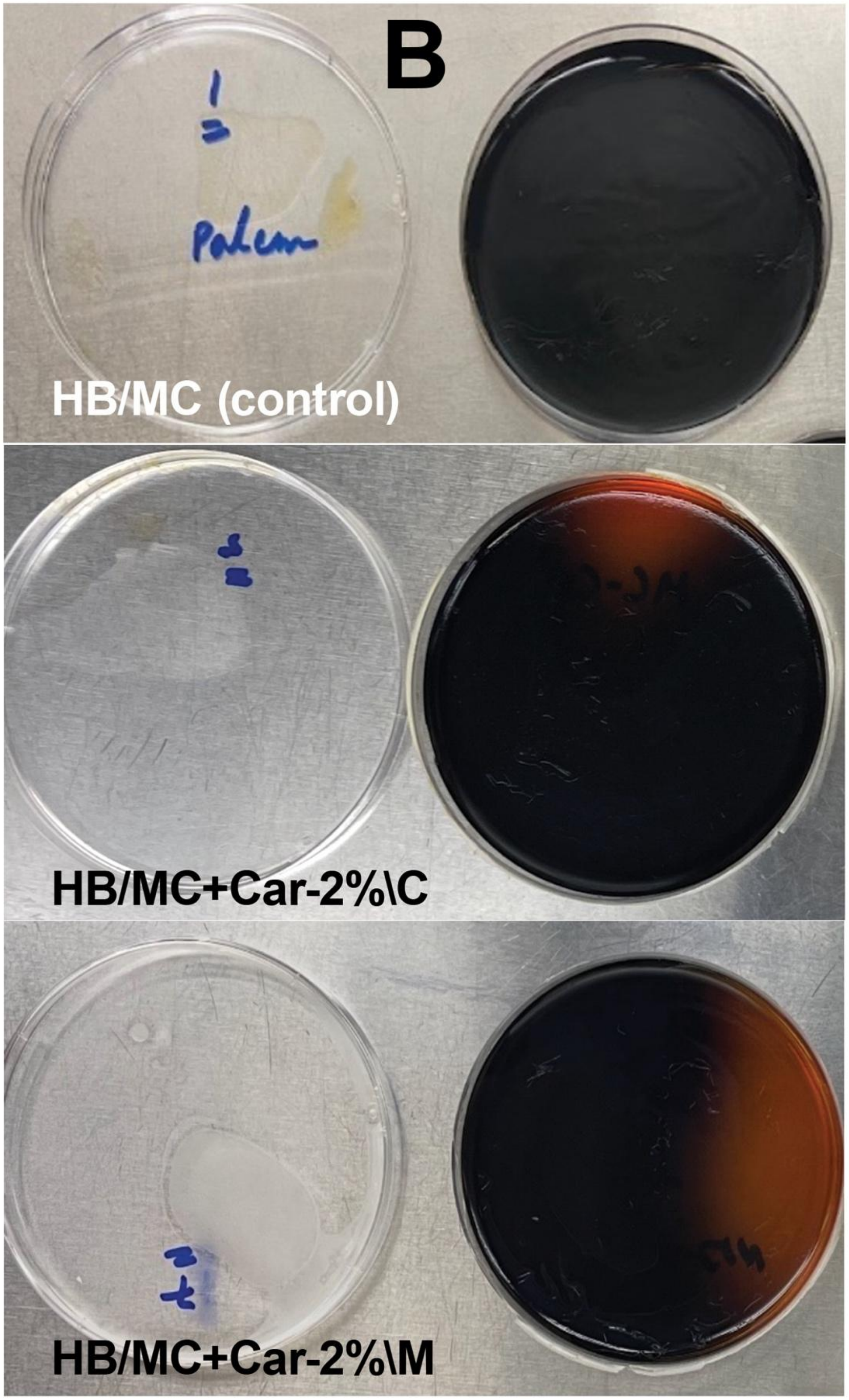
B
HB/MC (control)
HB/MC+Car-2%\C
HB/MC+Car-2%\M

Figure S2. Selected film samples illustrate the two methods used in this work to determine the antimicrobial activities of the HB/MC-based films against common foodborne pathogens. A: direct surface contact; B: film on lid (headspace release).
